# Modulatory Effect of 4-(methylthio)butyl Isothiocyanate Isolated From *Eruca Sativa* Thell. on DMBA Induced Overexpression of Hypoxia and Glycolytic Pathway in Sprague-Dawley Female Rats

**DOI:** 10.3389/fphar.2021.728296

**Published:** 2021-08-10

**Authors:** Davinder Singh, Sharad Thakur, Drishtant Singh, Harpal Singh Buttar, Balbir Singh, Saroj Arora

**Affiliations:** ^1^ Department of Botanical and Environmental Sciences, Guru Nanak Dev University, Amritsar, India; ^2^ Department of Molecular Biology and Biochemistry, Guru Nanak Dev University, Amritsar, India; ^3^ Department of Pathology and Laboratory Medicine, Faculty of Medicine, University of Ottawa, Ottawa, ON, Canada; ^4^ Department of Pharmaceutical Sciences, Guru Nanak Dev University, Amritsar, India

**Keywords:** 4-(methylthio)butyl isothiocyanate, breast cancer, hypoxia-inducible factor, glycolytic enzymes, 7,12-dimethylbenz [a] anthracene

## Abstract

4-(methylthio)butyl isothiocyanate (4-MTBITC) is a hydrolytic product from the plant *Eruca sativa* Thell. In the present study, we explored the anti-cancer effect of 4-MTBITC against 7,12-dimethylbenz [a] anthracene (DMBA) induced breast cancer. Hypoxic conditions were developed using a single dose of 60 mg/kg DMBA. Hepatic and renal parameters were increased along with antioxidants in cancer-bearing rats which were lowered with the treatment of 4-MTBITC. Further, it inhibited the up-regulation of glycolytic enzymes caused by DMBA. The hypoxia pathway was evaluated using RT-PCR and it was found that the 40 mg/kg doses of 4-MTBITC statistically lowered the expression of HIF-1α. Akt/mTOR signaling pathway was one of the major pathways involved in 4-MTBITC-induced cell growth arrest by western blotting. Amino acid profiling serum-free plasma revealed the downregulation of specific amino acids required for vital components of fast-growing cancer cells. 4-MTBITC reduced the levels of serine, arginine, alanine, asparagines, and glutamic acid. Histological examination also showed neoplastic growth following DMBA doses. 4-MTBITC treated rats showed less infiltration and normal physiology. Our findings for the first time demonstrated the potential therapeutic significance of 4-MTBITC on modulation of glycolytic enzymes and hypoxia pathway in female rats.

## Background

Many factors such as glycolytic enzymes and hypoxia-inducible factor-1α (HIF-1α) are known to be responsible for causing cancer. Increased glycolysis has been known as an indicator of cancer (Zhong et al., 1999). *Cancer* cells exhibit high aerobic glycolytic rates and produce high levels of lactate and pyruvate (Warburg effect). Lactate and pyruvate regulate hypoxia-inducible gene expression by stimulating the accumulation of HIF-1α (Lu et al., 2002). HIF-1α is a heterodimeric transcription factor that is a crucial regulator of the growing tumor’s response to hypoxia. Hypoxia may occur when aberrant blood vessels are shut down by becoming compressed or obstructed by growth (a common feature observed during the growth of tumors) and the cells that become a hypoxic shift to glycolytic metabolism (Powis and Kirkpatrick, 2004). HIF-1α overexpression is considered a marker of highly aggressive disease and has been detected in the brain, bladder, breast, colon, ovarian, pancreatic, renal and prostate tumors (Talks et al., 2000). Clinical data also indicate that HIF-1α overexpression is associated with a poor prognosis of hepatocellular carcinoma (Li et al., 2011). HIF-1α has been shown to activate hypoxia-responsive genes which are implicated in numerous aspects of tumorigenesis and cancer progression including proliferation, metabolism, angiogenesis, invasion and metastasis (Luo et al., 2014).

The functional gain of oncogenes and loss of function of tumor suppressor genes are the main characteristic of cancer cells (Hanahan and Weinberg, 2011). This causes uncontrolled proliferation to form a solid mass. So to maintain the energy level for dividing cells, a continuous supply of anabolic building blocks and energy carriers are established. Alterations of metabolic pathways were added to the six cancer hallmarks by Hanahan and Weinberg (Hanahan and Weinberg, 2011). These pathways contain up-regulation of glycolysis, mitochondrial biogenesis, lipid and amino acid metabolism, pentose phosphate pathway (PPP) and macromolecule biosynthesis (Singh et al., 2017).

7,12-dimethylbenz [a]anthracene (DMBA) is commonly employed as a model of polycyclic aromatic hydrocarbons (PAHs)–induced breast carcinogenesis due to its powerful carcinogenic and immunosuppressive effects (Jung et al., 2006). It causes carcinogenesis by causing or promoting mutations in the genes involved. When DMBA is given, it causes the cytosolic aryl hydrocarbon receptor (AhR) to be translocated into the nucleus, where it joins the AhR nuclear translocator (ARNT) protein to create an AhR/ARNT complex, which causes the cytochrome P450 enzyme to upregulate, metabolizing DMBA into an intermediate mutagenic epoxide that forms a DNA adduct that causes mutations (Trombino et al., 2000). Moreover, it also induces oxidative stress by generating reactive oxygen species (ROS) which plays a significant role in carcinogenesis (Karnam et al., 2017).

Understanding therapy resistance and successful treatment require identifying oncogenes and their related potential pathways. Through receptor tyrosine kinases, activated PI3K phosphorylates PIP2 to PIP3 (Thapa et al., 2015). Phosphorylation of Akt by PIP3 activates the mechanistic target of rapamycin (mTOR) which initiates signaling that stimulates cell growth and protein synthesis. As a result of the severity of cancer, we were compelled to look for an alternate supplement to treat cancer, as chemotherapy has several drawbacks. Dietary regimens and potent natural products are effective tools for lowering breast cancer mortality (Krishnamoorthy and Sankaran, 2016). Natural agents have recently gained a lot of interest due to their antioxidant and anticancer capabilities (Kaur et al., 2002; Kaur et al., 2005). Because 80% of the world’s population utilizes herbs to treat ailments in some form, WHO recommends using scientifically validated medicinal plants in primary health care after assessing quality, efficacy, and safety (Ekor, 2014).

Epidemiological studies demonstrate that the risk of cancer can be reduced significantly with the intake of cruciferous vegetables (Thimmulappa et al., 2002). This has been ascribed to the presence of secondary metabolites such as isothiocyanates (ITCs) from these plants which have the potential of cancer preventive activity (Verhoeven et al., 1996). These ITCs are hydrolytic products of glucosinolates (GSLs), which are most abundant in the reproductive parts of cruciferous plants in the maximum amount (Brown et al., 2003). Seeds of *Eruca sativa* Thell. also known as rocket salad contains a high amount of 4-(methylthio)butyl isothiocyanate (4-MTBITC) (Arora et al., 2018). Chemopreventive property of 4-MTBITC was documented for the first time in 1996 by Nastruzzi and others (Nastruzzi et al., 1996). Following this, a series of studies were done on different cancer cell lines (IMR-32, MCF-7, HeLa, MDA-MB-231, Caco-2, A549, HepG2, Hep3B, RT4, J82, UMUC3, etc) and different model organisms (swiss albino mice, SCID mice, Sprague Dawley rats) for *in-vivo* studies (Abbaoui et al., 2017; Prełowska et al., 2017). However, no report has been published on the modulatory effect of 4-MTBITC on differential expression of the glycolytic enzymes under hypoxic conditions in mammary tissues.

In the present study, we sought to demonstrate if the 4-MTBITC could act as a novel anti-tumor agent by altering the glycolytic pathway and hypoxia-regulating genes in breast cancer-bearing rats.

## Materials and Methods

### Isolation and Characterization

4-MTBITC was isolated from the seeds of plant *Eruca sativa* using the method described by Arora et al. (2016) with some modifications. The isolated compound was analyzed on UHPLC (Shimadzu, Kyoto, Japan).

### Ethical Statement

The rats were housed in the Central Animal Facility under standard animal husbandry conditions. The experimental protocol was approved by the Animal Ethical Committee of Guru Nanak Dev University (GNDU), Amritsar. The rules and regulations of control and supervision of experimental animals (CPCSEA) were followed according to the Ministry of Environment and Forests (File No. 226/CPCSEA/2014/17), Government of India. All experiments were performed following ethical standards.

### Animal Study

#### Experimental Animal Model

Twenty-four female Sprague-Dawley rats were obtained from the National Institute of Pharmaceutical Education and Research, Mohali, Punjab. After 2 weeks of acclimatization, rats were divided randomly into four groups (n = 6). Initially twelve rats were administered with a single 60 mg/kg dose of DMBA, a dose sufficient to make 100% tumor incidence in the control group (Kumar et al., 2017; Sheokand, Navik and Bansal, 2019). After 90 days of DMBA administration when tumor volume measured about 20 mm^3^ the animals were divided in experimental groups as follows: group A (administered orally with sunflower oil), group B (breast cancer-induced rats), group C (administered orally with 40 mg/kg 4-MTBITC in sunflower oil for the duration same as in group D), group D (breast cancer-induced rats were treated with 40 mg/kg oral doses of 4-MTBITC for 1 month on alternative days). 4-MTBITC was gavaged for total 15 days in 1 month. 40 mg/kg dose was selected from acute and subchronic toxicity studies and duration of repeated dose was selected from pharmacokinetic studies. Rats were palpated every day for mammary tumors. The quantitative measurements were done using caliper scale and equation: π/6(a)^2^*(b). Throughout the treatment, animals were observed for morbidity or mortality. The body weight of the animals was recorded before the start of the experiment, weekly during the experiment, and at the end of the experiment.

### Biochemical Analyses

Blood samples were collected on the last day of the studies from the retro-orbital plexus and centrifuged at 3,000 rpm for 15 min. The serum was stored at -80°C for further analysis. Serum aliquots were used to estimate the different biochemical parameters like serum glutamic oxaloacetic transaminase (SGOT), serum glutamic pyruvic transaminase (SGPT), direct bilirubin, total bilirubin, creatinine, triglycerides, and cholesterol using readymade ERBA kits.

The weighed samples of liver were homogenized in ice-cold Tris-HCl buffer (pH 8.0) for 5 min and centrifuged at 8,000 rpm for 15 min at 4°C. The supernatant was stored at -80°C for enzyme analysis. Superoxide dismutase (SOD) was measured according to the method of Marklund and Marklund (1974). The method is based on the ability of the SOD enzyme to inhibit the phenazine methosulphate mediated reduction in nitroblue tetrazolium dye. Reduced glutathione levels were assessed according to the method of (Moron et al. (1979). The method is based on the reduction of 5,5′-dithiobis (2-nitrobenzoic acid) (DTNB) with glutathione to produce a yellow color. MDA content was estimated according to the method provided by Ohkawa et al. (1979). Hydroxyl radicals were quantified according to Gutteridge et al. (1981). Hydroxyl radicals were estimated by their reaction with 2-deoxy ribose resulting in the formation of TBA reacting species.

### RNA Isolation and Quantitative Real-Time Polymerase Chain Reaction

Total RNA was isolated using TRIzol (Thermo Fisher). Briefly, 100 mg frozen tissue was weighed and minced in pestle mortar using liquid nitrogen. Instantly, TRIzol was added and centrifuged. Chloroform was used to induce phase separation. Isopropanol was used to precipitate the RNA. Isolated RNA was washed thrice with 70% ethanol. DNA was removed using DNase treatment. The extracted RNA was quantified and qualified using a NanoDrop spectrophotometer. Finally, total RNA was normalized to avoid false changes in gene expression. Using Luna One-Step RT-qPCR (New England BioLabs), the gene expression of hexokinase, phosphoglucose isomerase, aldolase, triosephosphate isomerase, glyceraldehyde 3-phosphate dehydrogenase (GAPDH), phosphoglycerate isomerase, phosphoglycerate mutase, enolase, pyruvate kinase, HIF-1α, HIF-1β, prolyl hydroxylase (PHD), mTOR, phosphatase and tensin homolog (PTEN), Heat shock protein-90α (HSP-90α), and succinate dehydrogenase was recorded. Primer sequences are enlisted in Supplementary Table S1. The relative mRNA expression of each target was normalized to 18 S.

### Estimation of Lactate Content and Other Enzymes

Lactate content was estimated using YSI 1500 L sport electron probe according to the manufacturer’s instructions. Briefly, 25 μL whole blood was injected into an automated YSI 1500 L unit for immediate analysis. Lactate dehydrogenase was estimated by measuring the reduction of NADH into NAD^+^ according to the protocol of King (King, 1965). The activity of fructose-1,6-bisphosphatase (FBPase) was measured according to the method of Taketa and Pogell and 6-phosphogluconate dehydrogenase (6PGD) was measured spectrophotometrically according to the method given by Ben-Bassat and Goldberg (Taketa and Pogell, 1965; Ben-Bassat and Goldberg, 1980).

### Western Blot Analysis

Total protein in breast tissue homogenate was estimated using a readymade total protein estimation kit (ERBA). Approximately 40 μg protein was loaded on each well of polyacrylamide gel and separated by SDS-PAGE. The resolved proteins were transferred to the polyvinylidene difluoride membrane (0.2 μm) by electrophoresis. Then the membranes were blocked by 5% milk powder in 50 mM Tris-HCL, 150 mM NaCl, and 0.1% Tween 20 at room temperature for 1 h. The membrane was then immunoblotted for overnight at 4°C with either a rabbit monoclonal anti-HIF-1α (Cell Signaling Technology, United States), a rabbit polyclonal anti-Akt (Cell Signaling Technology, United States), a rabbit polyclonal anti-mTOR (Cell Signaling Technology, United States), a rabbit polyclonal anti-TNF-α (Tumor necrosis factor-α) (Cell Signaling Technology, United States), p21 (Cell Signaling Technology, United States), a rabbit monoclonal anti-PHD-2/Egln1 (Cell Signaling Technology, United States), a rabbit monoclonal anti-p53 (Cell Signaling Technology, United States), a rabbit monoclonal anti-NF-kB-p65 (Nuclear factor-kB) (Cell Signaling Technology, United States) or a rabbit polyclonal anti-β-Actin (Cell Signaling Technology, United States) diluted at 1/1,000–1/10,000 in TBS tween 0.1%. Subsequently, membranes were washed thrice with 50 mM phosphate-buffered saline and 0.1% tween-20. Following incubation with horseradish peroxidase-conjugated anti-rat secondary antibody diluted at 1/1,000 for 1 h at room temperature. The blots were developed using ECL chemilluminicance substrate solution. Autoradiographic signals were captured on a Genegenious imaging system using the Genesnap software.

### Enzyme-Linked Immunosorbent Assay

For the estimation of adiponectin, leptin, insulin, and IL6, fresh plasma was used. Respective rat ELISA kits (RayBiotech, United States) were used according to the manufacturer’s instructions. 96 well Elisa plate was read on a multimode microplate reader (Sunergy HT, BioTek).

### Amino Acid Analysis

#### Sample Preparation

Samples (liver) were prepared by digesting tissue homogenates in 6 N HCl along with 0.1% phenol in tightly closed 25 ml reagent bottles at 110°C for 24 h. Further, the samples were dried using a vacuum evaporator (Buchi) and reconstituted in 0.1 mol/L HCl solution for analysis. A total of 19 amino acid standards were analyzed including aspartic acid, glutamic acid, asparagines, serine, histidine, glutamine, glycine, threonine, arginine, alanine, tyrosine, cysteine, valine, methionine, tryptophan, phenylalanine, isoleucine, leucine, and lysine and their linearity of response across six-point serial dilution was plotted.

#### Chromatographic System

The HPLC-based amino acid analyzer consisted of Nexera X2, Shimadzu coupled with Shimadzu RF-20A prominence fluorescence detector was used. The HPLC separation of derivatized amino acids required the two mobile phases. Mobile phase A consists of 20 mmol/L potassium phosphate buffer (pH 6.5) and mobile phase B consists of 45:40:15 acetonitrile:methanol: water. All buffers were filtered through 0.2 µm filtered and degassed by sonication. The derivatized amino acids were separated on a Phenomenex Luna C18 column (250 mm Χ 4.6 mm id., 5 µm particle size). The chromatographic separation was performed at 35°C. Fluorometric measurement was conducted with an excitement wavelength of 350 nm and an emission wavelength of 450 nm and a gain at 100. The flow rate of the mobile phase was 0.95 ml/min throughout the analysis and sample injection volume for derivatization was 1 µL. The gradient conditions were as follows: initial conditions are 2% mobile phase A; from time 0.01–2 min the gradient changes to 90% solvent A and 10% solvent B; from 2 to 33.40 min the gradient changes to 43% solvent A and 51% solvent B; from 33.40 to 33.50 min the gradient changes to 100% solvent A and 0% solvent B; from 33.50 to 39.30 min the gradient remains same as 100% solvent A and 0% solvent B; from 39.30 to 39.40 min the gradient changes to 90% solvent A and 10% solvent B; from 39.4 to 45 min the gradient was maintained to 90% solvent A and 10% solvent B.

The HPLC run time for the separation of the derivatized amino acids in the sample or standard is 50 min. The amino acid derivatization reagents were prepared freshly each day. *O*-phthalaldehyde (OPA) was prepared by dissolving 10 mg of OPA in 0.3 ml methanol and mixed completely by sonication. Then 0.7 ml borate buffer and 4 ml HPLC grade water were added to make a working solution. Mercapto propionic acid (MPA) was prepared by adding 10 µL in 10 ml of 0.1 mol/L borate buffer. 9-fluorenylmethyl chloroformate (FMOC) was prepared by adding 4 mg into 20 ml of acetonitrile. The derivatization of amino acids in the samples and standards was performed in an automated fashion using a Nexera SIL-30AC autosampler kept at 15°C. The needle of the autosampler was rinsed with 50% methanol/water between injections. During chromatography of the sample, the derivatization of the next sample is started so that there is no delay in the injection of the next sample.

### Histopathology

At the time of scheduled euthanasia, all animals underwent a full gross necropsy examination. Mammary tissue and tumor masses were preserved in 10% neutral buffered formalin (NBF) for each rat. All collected tissues were examined for histopathological changes with hematoxylin and eosin (H&E) staining after full fixation in 10% NBF. The tissues were trimmed, embedded in paraffin, sectioned, mounted on microscope slides, and stained with hematoxylin and eosin. The paraffin-embedded blocks were sectioned 3 μm thin and stained with H&E.

### Statistical Analysis

Data were analyzed statistically using a one-way analysis of variance (ANOVA) in GraphPad Prism 8. All results are presented as mean ± SEM from at least three independent experiments. Dunnett’s multiple comparison test was used to compare multiple groups. For amino acid analysis ANOVA with sidak multiple comparison test was used. A difference was considered significant at the *p* < 0.05 level.

## Results

### Metabolic Parameters

**FIGURE 1:**
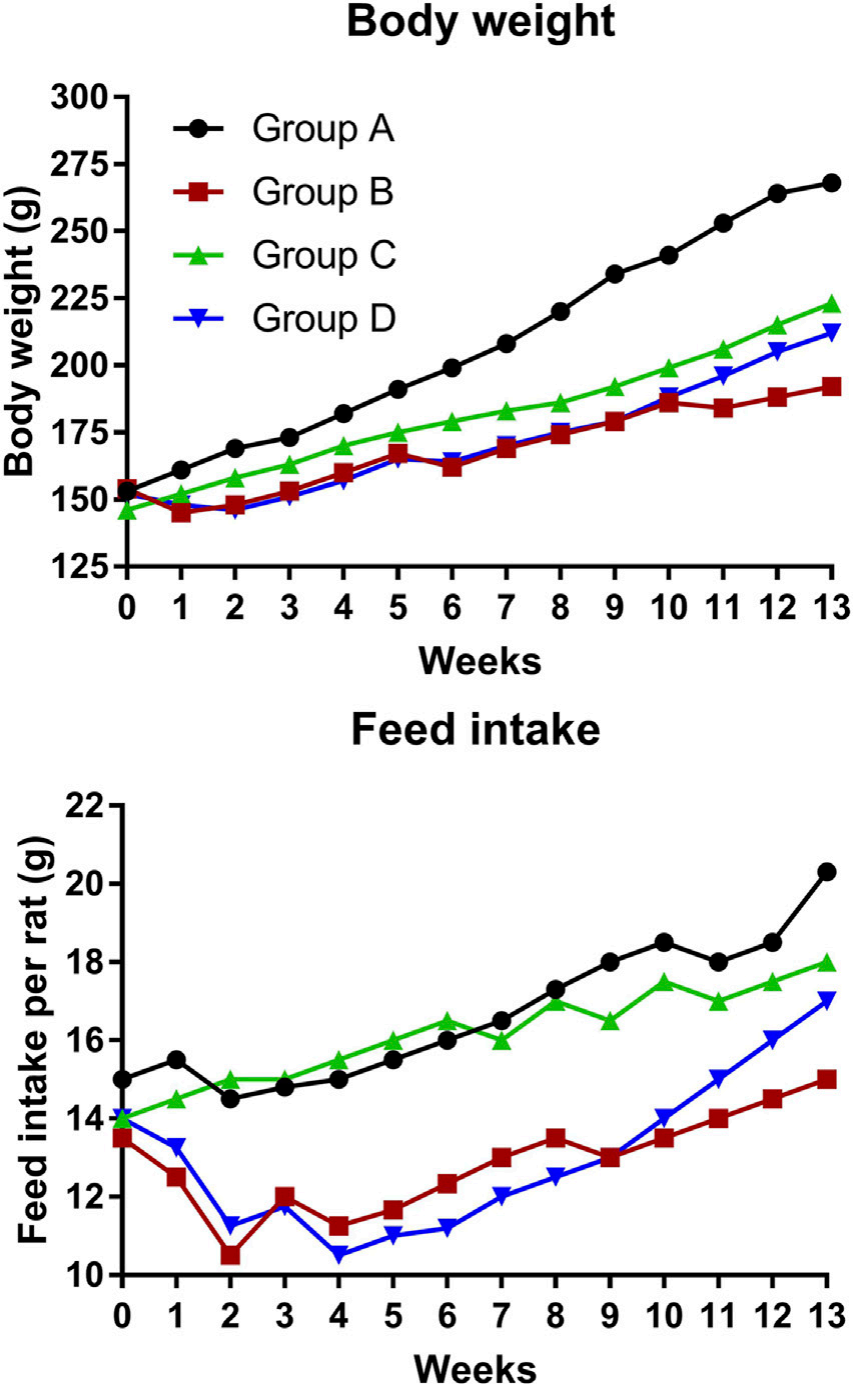
Bodyweight transitions and feed intake changes during the animal study.

Figure 1 depicts the effect of 4-MTBITC on body weight and feed intake. In group D, the body weight was gained after the treatment of 4-MTBITC as compared to group B, the feed intake was also observed to increase after the treatment of 4-MTBITC.

### Hepatic, Renal, and Lipid Profile

Serum parameters were recorded for all the rats. It was found that the parameters for hepatic (SGOT (161% at *p* < 0.01), SGPT (87% at *p* < 0.01), direct bilirubin (133% at *p* < 0.01), total bilirubin (58% at *p* < 0.01)), renal (creatinine (71% at *p* < 0.01)), and lipid profile (triglyceride (63% at *p* < 0.01), cholesterol (115% at *p* < 0.01)) were significantly enhanced in rats treated with DMBA (group B) compared to control group. In group D the levels of SGOT were brought down by 59% (*p* < 0.01), SGPT by 30% (*p* < 0.01), direct bilirubin by 29% (*p* < 0.01), total bilirubin by 26% (*p* < 0.01), creatinine by 27% (*p* < 0.01), triglyceride by 36% (*p* < 0.01) and cholesterol by 30% (*p* < 0.01) compared to group B (Table 1).

**TABLE 1:** Effect of 4-(methylthio)butyl isothiocyanate on hepatic, renal, and lipid profile in control and experimental group of animals. SGOT: Serum glutamic oxaloacetic transaminase, SGPT: Serum glutamic pyruvic transaminase. All the values are represented as mean ± SEM for six animals. Significant levels are **p < 0.01 when compared with group A and ##*p* < 0.01 when compared with group B.

Parameters	Group A	Group B	Group C	Group D	% change in group B compared to group A	% change in group B compared to group A
SGOT (IU/L)	25.66 ± 1.69	67.05 ± 2.69**	27.69 ± 1.43	27.17 ± 1.28##	161	−59
SGPT (IU/L)	53.66 ± 2.93	100.33 ± 5.65**	59.54 ± 5.23	70.36 ± 4.72##	87	−30
Direct bilirubin (mg/dl)	0.03 ± 0.001	0.07 ± 0.003**	0.05 ± 0.004	0.05 ± 0.002##	133	−29
Total bilirubin (mg/dl)	0.12 ± 0.01	0.19 ± 0.01**	0.12 ± 0.01	0.14 ± 0.01##	58	−26
Creatinine (mg/dl)	0.94 ± 0.05	1.61 ± 0.06**	0.99 ± 0.1	1.18 ± 0.1##	71	−27
Triglyceride (mg/dl)	65.12 ± 3.89	106.48 ± 4.79**	60.73 ± 2.42	67.63 ± 3.64##	63	−36
Cholesterol (mg/dl)	48.17 ± 4.83	103.58 ± 4.06**	57.77 ± 7.82	72.24 ± 2.93##	115	−30
Total Protein (mg/g tissue)	392.25 ± 15.69	300.02 ± 9.53**	357.71 ± 16.36	342.97 ± 22.19	23	−14

### Oxidative Stress Parameters

Table 2 depicts the effect of DMBA and 4-MTBITC on antioxidant levels in experimental animals. In group B the activity of SOD was decreased by 33% (*p* < 0.05) and levels of reduced glutathione by 17% (*p* < 0.01) compared to normal rats. On the other hand, in group D the activity of SOD was increased by 112% (*p* < 0.01) and the levels of glutathione were increased by 112% (*p* < 0.01) compared to group B. DMBA treatment markedly increased the levels of MDA (122% at *p* < 0.01) and hydroxyl levels (73% at *p* < 0.01) in group B compared to group A. Oral doses of 4-MTBITC reduced the levels of MDA by 31% (*p* < 0.01) and hydroxyl radicals by 30% (*p* < 0.01) in group D compared to group A.

**TABLE 2:** Effect of 4-(methylthio)butyl isothiocyanate on enzymatic and non-enzymatic antioxidant levels in control and experimental group of animals. SOD: Superoxide dismutase. All the values are represented as mean ± SEM for six animals. Significant levels are *p < 0.05, **p < 0.01 when compared with group A and ##*p* < 0.01 when compared with group B.

Parameters	Group A	Group B	Group C	Group D	% change in group B compared to group A	% change in group D compared to group B
SOD (IU/min/mg protein)	4.70 ± 0.30	3.13 ± 0.36*	5.48 ± 0.38	6.64 ± 0.36##	−33	112
Reduced glutathione (µM/g tissue)	417.98 ± 21.87	242.18 ± 10.48**	348.72 ± 26.44	514.34 ± 35.20##	−17	112
MDA (nM of MDA released/mg protein)	0.55 ± 0.05	1.22 ± 0.13**	0.53 ± 0.04	0.84 ± 0.07##	122	−31
Hydroxyl radicals (nmol/g tissue)	2.40 ± 0.17	4.16 ± 0.28**	2.34 ± 0.20	2.89 ± 0.14##	73	−30

### 3.4. Modulation of Glycolytic Pathways

**FIGURE 2:**
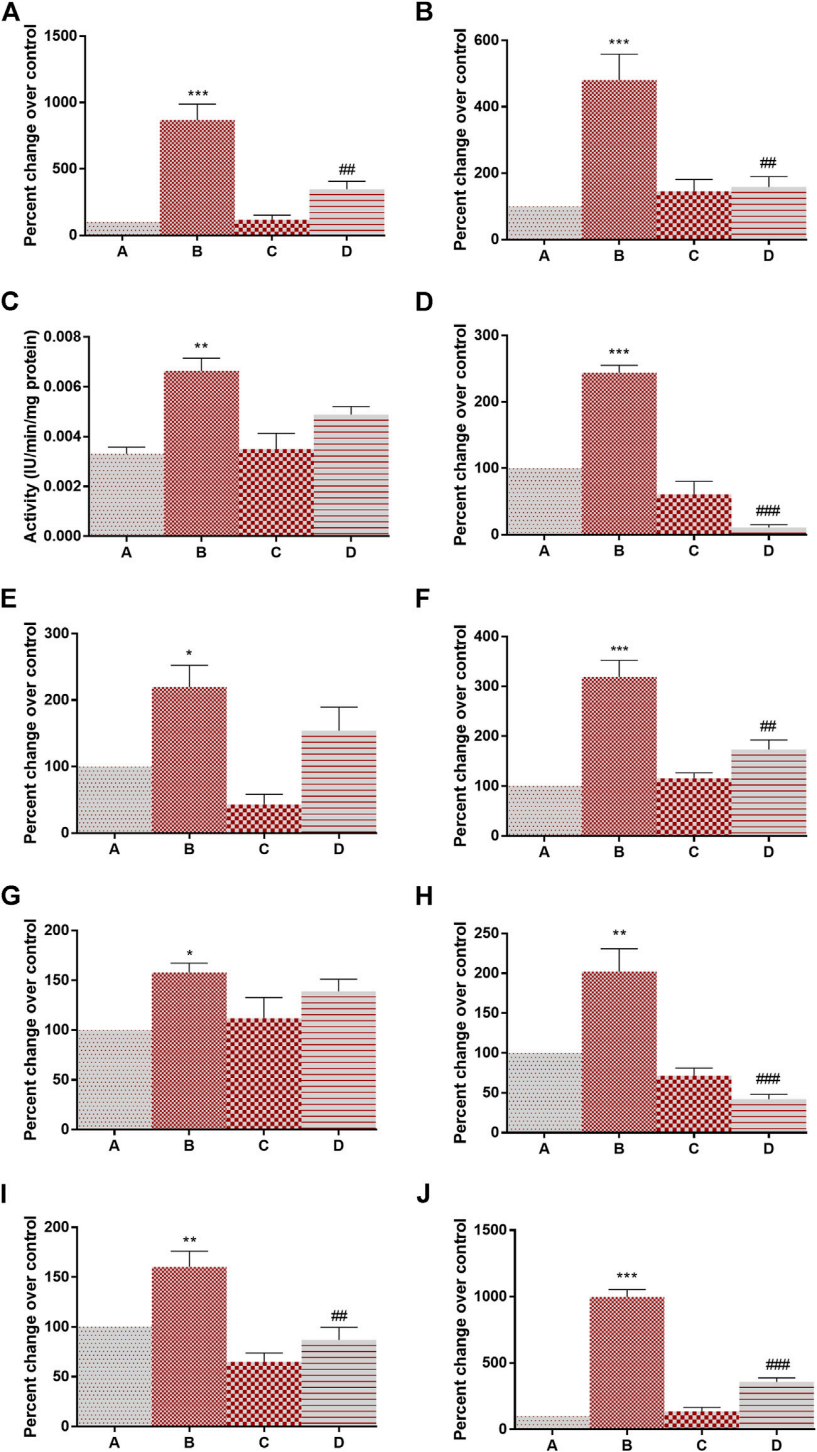
Effect of 4-(methylthio)butyl isothiocyanate on gene expression analysis of glycolytic enzymes in the control and experimental group of animals. **(A)** Hexokinase, **(B)** Phosphoglucose isomerase, **(C)** phosphofructokinase, **(D)** Aldolase, **(E)** Triosphosphate isomerase, **(F)** Glyceraldehyde 3-phosphate dehydrogenase, **(G)** Phosphoglycerate kinase, **(H)** Phosphoglycerate mutase, **(I)** Enolase, **(J)** Pyruvate kinase. All the values are represented as mean ± SEM (n = 3). Significant levels are *p < 0.05, **p < 0.01, ***p < 0.001 when compared with group A and #*p* < 0.05, ##*p* < 0.01 when compared with group B.

Figure 2 depicts the effect of DMBA and 4-MTBITC on gene expression analysis of glycolytic enzymes. The percent change in mRNA expression of hexokinase (768% at *p* < 0.001), phosphoglucose isomerase (380% at *p* < 0.001), phosphofructokinase (76% at *p* < 0.01), aldolase (143% at *p* < 0.001), triosephosphate isomerase (119% at *p* < 0.05), GAPDH (219% at *p* < 0.001), phosphoglycerate kinase (57% at *p* < 0.02), phosphoglycerate mutase (102% at *p* < 0.01), enolase (60% at *p* < 0.01), and pyruvate kinase (897% at *p* < 0.001) was significantly increased after the treatment of DMBA as compared to control. The counter-treatment of 4-MTBITC significantly decreased the gene expression of hexokinase (59% at *p* < 0.01), phosphoglucose isomerase (66% at *p* < 0.01), aldolase (54% at *p* < 0.001), GAPDH (45% at *p* < 0.01), phosphoglycerate mutase (79% at *p* < 0.001), enolase (45% at *p* < 0.01) and pyruvate kinase (64% at *p* < 0.001) as compared to group B. 4-MTBITC treatment in group D non-significantly decreased the percent change in gene expression of phosphofructokinase (26%), triosephosphate isomerase (29%) and phosphoglycerate kinase (11%) as compared to group B.

### Hypoxia Pathway Evaluation

**FIGURE 3:**
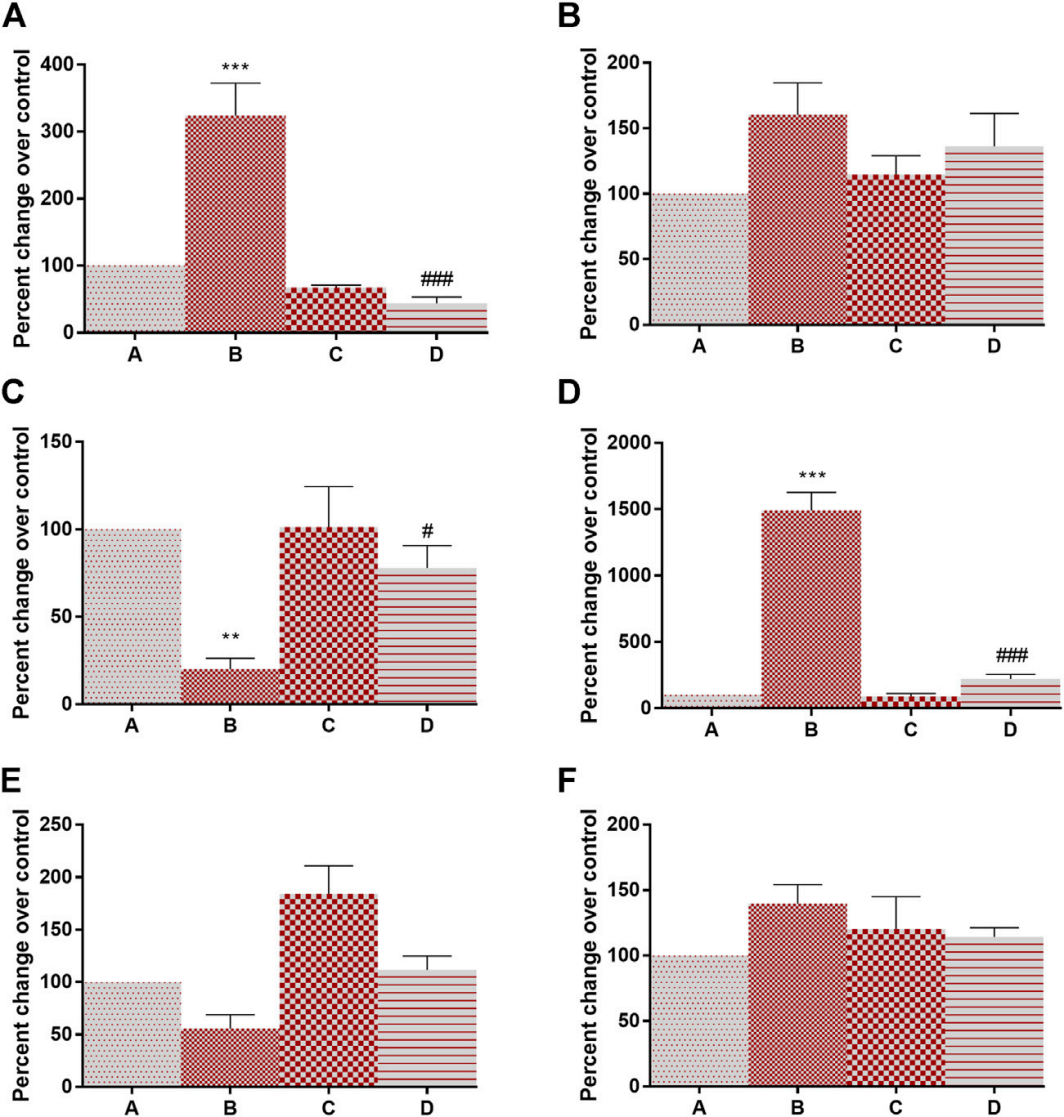
Gene expression analysis of hypoxia-inducible factor-associated genes in control and experimental group of animals. **(A)** Hypoxia inducible factor-1α, **(B)** Hypoxia inducible factor-1β, **(C)** Prolyl hydroxylase, **(D)** Mammalian target of rapamycin, **(E)** phosphatase and tensin homolog, **(F)** Heat shock protein-90α. All the values are represented as mean ± SEM (n = 3). Significant levels are **p < 0.01, ***p < 0.001 when compared with group A and #*p* < 0.05, ###*p* < 0.001 when compared with group B.

Having determined that 4-MTBITC disrupts the glycolytic pathway, we wanted to determine if 4-MTBITC attenuated hypoxia-inducible transcription. Evaluation of HIF-1α, HIF-1β, PHD, mTOR, PTEN, HSP-90α mRNA expression was done using RT-PCR. There a significant overexpression of HIF-1α (223% at *p* < 0.001) and mTOR (1,391% at *p* < 0.001) and downregulation in PHD (79% at *p* < 0.01) was observed after the treatment of DMBA in group B as compared to group A while an insignificant increase in HIF-1β (60%) and HSP-90α (39%) and decrease in PTEN (44%) was observed. Following the treatment of 4-MTBITC in group D the upregulated expression of HIF-1α (86% at *p* < 0.001), and mTOR (5% at *p* < 0.001) was lowered while PHD (443% at *p* < 0.05) was upregulated (Figure 3). Moreover, HIF-1β (15%), PTEN (101%), and HSP-90α (18%) were insignificantly changed.

### Modulation of Lactate Production and Other Enzymes

**FIGURE 4:**
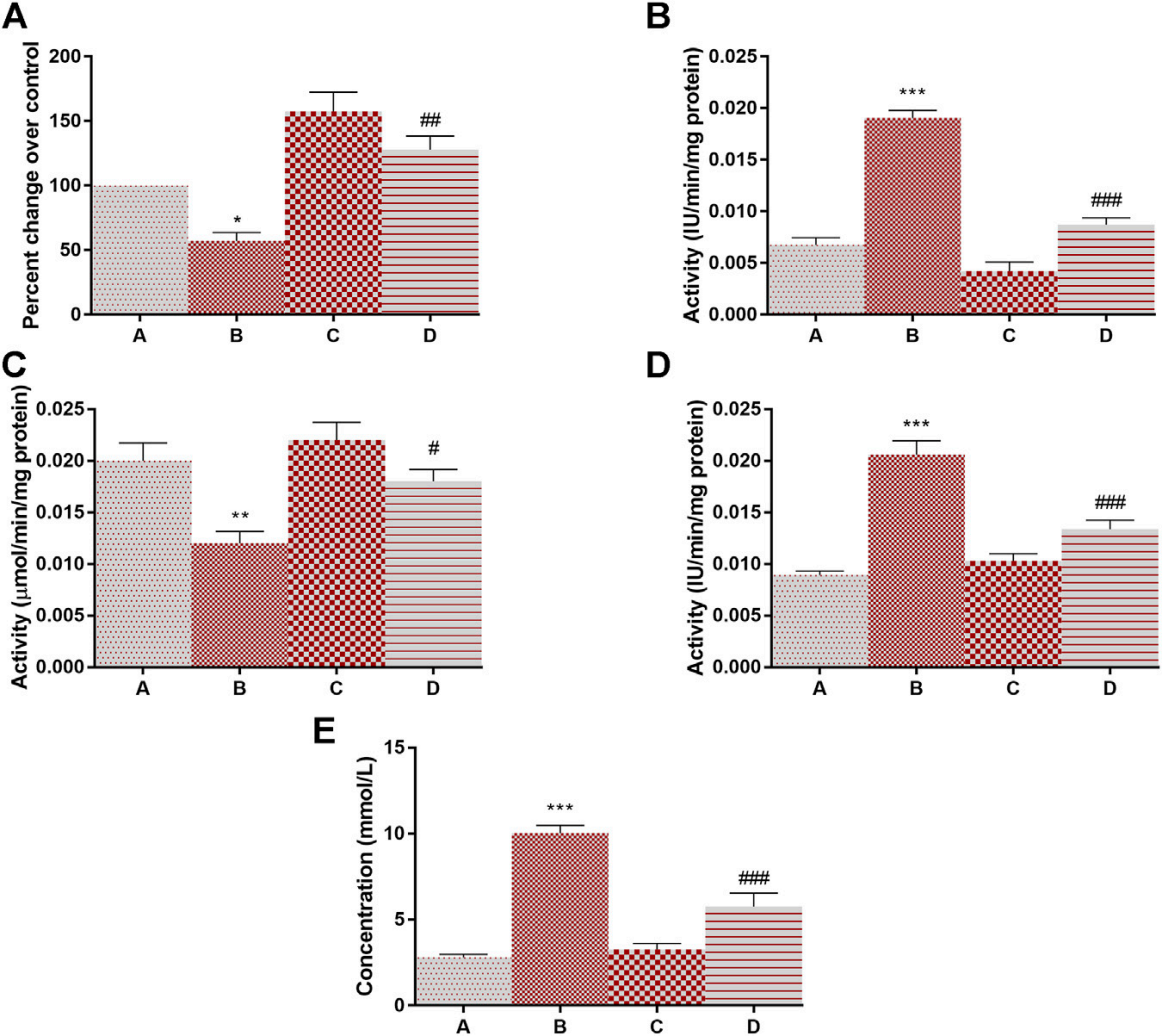
The activity of succinate dehydrogenase, lactate dehydrogenase, fructose-1,6-bisphosphatase, phosphogluconate dehydrogenase, and levels of lactate content in response to 4-(methylthio)butyl isothiocyanate and DMBA. **(A)** Succinate dehydrogenase, **(B)** Lactate dehydrogenase, **(C)** Fructose-1,6-bisphosphatase, **(D)** Phosphogluconate dehydrogenase, **(E)** Lactate content. All the values are represented as mean ± SEM (n = 3). Significant levels are *p < 0.05, **p < 0.01, ***p < 0.001 when compared with group A and #*p* < 0.05, ##*p* < 0.01, ###*p* < 0.001 when compared with group B.

The activity of lactate dehydrogenase (145% at *p* < 0.001) and 6-PGD (138% at *p* < 0.001) was increased in DMBA treated rats as compared to group A. Following the treatment of 4-MTBITC in group D, the activity of lactate dehydrogenase was decreased by 59% at *p* < 0.001 and 6-PGD by 48% at *p* < 0.001 as compared to group B (Figures 4A,B). Succinate dehydrogenase, an essential enzyme of the tricarboxylic acid cycle (TCA cycle), was found to be significantly downregulated (42% at *p* < 0.05) in group B as compared to group A (Figure 4C). 4-MTBITC treatment increase the expression of succinate dehydrogenase by 123% at *p* < 0.01 as compared to group D. The activity of FBPase responsible for conversion of fructose 1,6-bisphosphate to fructose 6-phosphate in gluconeogenesis for storage of glucose moieties was observed to be significantly decreased (40% at *p* < 0.01) under hypoxic conditions of group B compared to group A (Figure 4D). A significant increase (50% at *p* < 0.05) was observed in group D as compared to group B following the treatment of 4-MTBITC. Furthermore, the lactate content was quantified in all the experimental animals and it was found that the DMBA increased the lactate levels (214% at *p* < 0.001) which were then significantly reduced (27% at *p* < 0.001) by 4-MTBITC in group D as compared to group B (Figure 4E).

### Inflammation and Glucose Regulation

**FIGURE 5:**
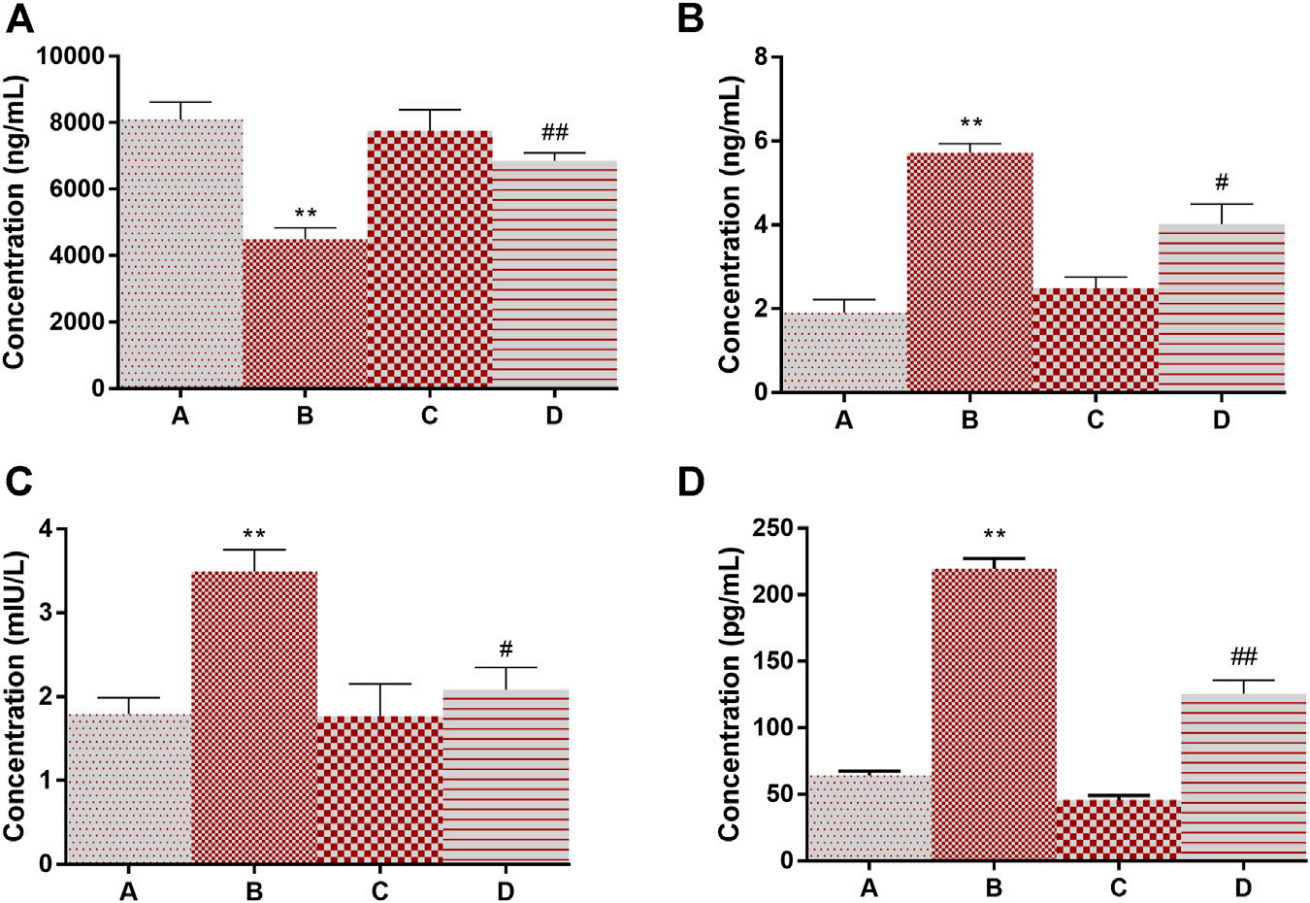
Modulation of levels of adiponectin, leptin, insulin, and IL6 in response to 4-(methylthio)butyl isothiocyanate and DMBA. **(A)** Adiponectin, **(B)** Leptin, **(C)** Insulin, **(D)** IL-6. **p < 0.01 when compared with group A and #*p* < 0.05, ##*p* < 0.01 when compared with group B.

Adiponectin is known for its overall energy homeostasis and metabolism. Significant elevation (200% at *p* < 0.01) in serum leptin and a decrease in adiponectin levels (44% at *p* < 0.01) were observed in group B compared to vehicle control rats (Figures 5A,B). 4-MTBITC treatment attenuated DMBA induced alteration in leptin (29% at *p* < 0.05) and adiponectin (52% at *p* < 0.01) levels compared to group B. It was observed that the levels of insulin were significantly increased (94% at *p* < 0.01) in cancer-bearing rats as compared to control (Figure 5C). Further, the treatment of 4-MTBITC (group D) reduced (40% at *p* < 0.05) the insulin significantly from group B. IL-6 a pleiotropic cytokine related to inflammation was seen significantly enhanced (241% at *p* < 0.01) in group B as compared to the control group (Figure 5D). 4-MTBITC was able to reduce (42% at *p* < 0.01) the IL-6 levels significantly as compared to group B.

### Modulation of Amino Acid Profile

The mean normalized amino acid concentrations of all the groups were investigated (Supplementary Figure S2). The average levels of serine (169% at *p* < 0.001), arginine (37% at *p* < 0.05), alanine (81% at *p* < 0.001), asparagine (43% at *p* < 0.01) and glutamic acid (145% at *p* < 0.001) were increased in animals bearing cancer (group B). Treatment of 4-MTBITC in group D decreased the levels of serine by 31% (*p* < 0.05), arginine by 54% (*p* < 0.001), alanine by 43% (*p* < 0.001), asparagines by 26% (*p* < 0.05) and glutamic acid by 24% (*p* < 0.05) as compared to group B (Table 3). On the other hand, the concentrations of tryptophan (40% at *p* < 0.01) were decreased in response to a carcinogen (group B) and increased by 4-MTBITC (53% at *p* < 0.05).

**TABLE 3:** Concentration (nmol/ml) of recorded amino acids in different groups. Data represents mean ± SEM. One way ANOVA with sidak multiple comparison test was applied. Significant levels are *p < 0.05, **p < 0.01, ***p < 0.001 when compared with group A and #*p* < 0.05, ###*p* < 0.01 when compared with group B.

Amino acids	Group A	Group B	Group C	Group D	% change in group B compared to group A		% change in group D compared to group B
Glutamine	4.98 ± 0.33	4.64 ± 0.43	4.9 ± 0.36	5.8 ± 0.35	−6		25
Threonine	8.28 ± 0.19	8.59 ± 0.07	7.85 ± 0.05	8.61 ± 0.46	4		−0.2
Alanine	10.21 ± 0.71	18.55 ± 0.79***	7.32 ± 0.34	10.59 ± 1.22###	81		−43
Arginine	11.31 ± 0.6	15.54 ± 1.39*	11.96 ± 0.36	7.19 ± 0.74###	37		−54
Asparagine	6.26 ± 0.34	8.95 ± 0.65**	5.48 ± 0.13	6.65 ± 0.64#	43		−26
Aspartic Acid	11.58 ± 0.34	10.12 ± 0.31	12.92 ± 0.88	11.38 ± 0.6	−13		12
Glutamic Acid	6.4 ± 0.39	15.68 ± 1.72***	10.09 ± 0.4	11.83 ± 0.71#	145		-24
Glycine	8.72 ± 0.34	8.31 ± 0.29	8.3 ± 0.05	8.01 ± 0.11	−5		−4
Histidine	6.35 ± 0.25	7.84 ± 0.42	7.79 ± 0.02	9.36 ± 0.75	23		19
Valine	5.33 ± 0.65	7.17 ± 0.22	5.1 ± 0.54	8.74 ± 0.99	34		22
Isoleucine	10.13 ± 0.06	8.09 ± 0.1	7.84 ± 1.12	7.28 ± 0.48	−20		−10
Methionine	14.91 ± 0.36	13.68 ± 0.61	14.41 ± 0.36	14.34 ± 0.52	−8		5
Phenylalanine	9.01 ± 0.34	7.91 ± 0.46	7.69 ± 0.07	8.83 ± 0.08	−12		12
Serine	4.44 ± 0.32	11.93 ± 1.32***	4.08 ± 1.2	8.21 ± 0.46#	169		−31
Tryptophan	13.79 ± 0.65	8.22 ± 0.60**	11.04 ± 1.68	12.61 ± 0.75#	−40		53
Tyrosine	18.51 ± 0.04	17.54 ± 0.54	17.08 ± 1.36	17.87 ± 0.07	−5		2
Cysteine	8.09 ± 0.36	8.15 ± 0.06	8.41 ± 0.03	8.69 ± 0.34	0.7		7
Leucine	6.31 ± 0.59	7.9 ± 0.59	7.42 ± 1.22	6.37 ± 0.34	25		−19
Lysine	2.39 ± 0.53	4.33 ± 0.58	4.04 ± 0.78	4.57 ± 0.35	−69		5

### Protein Expression Analysis

**FIGURE 6:**
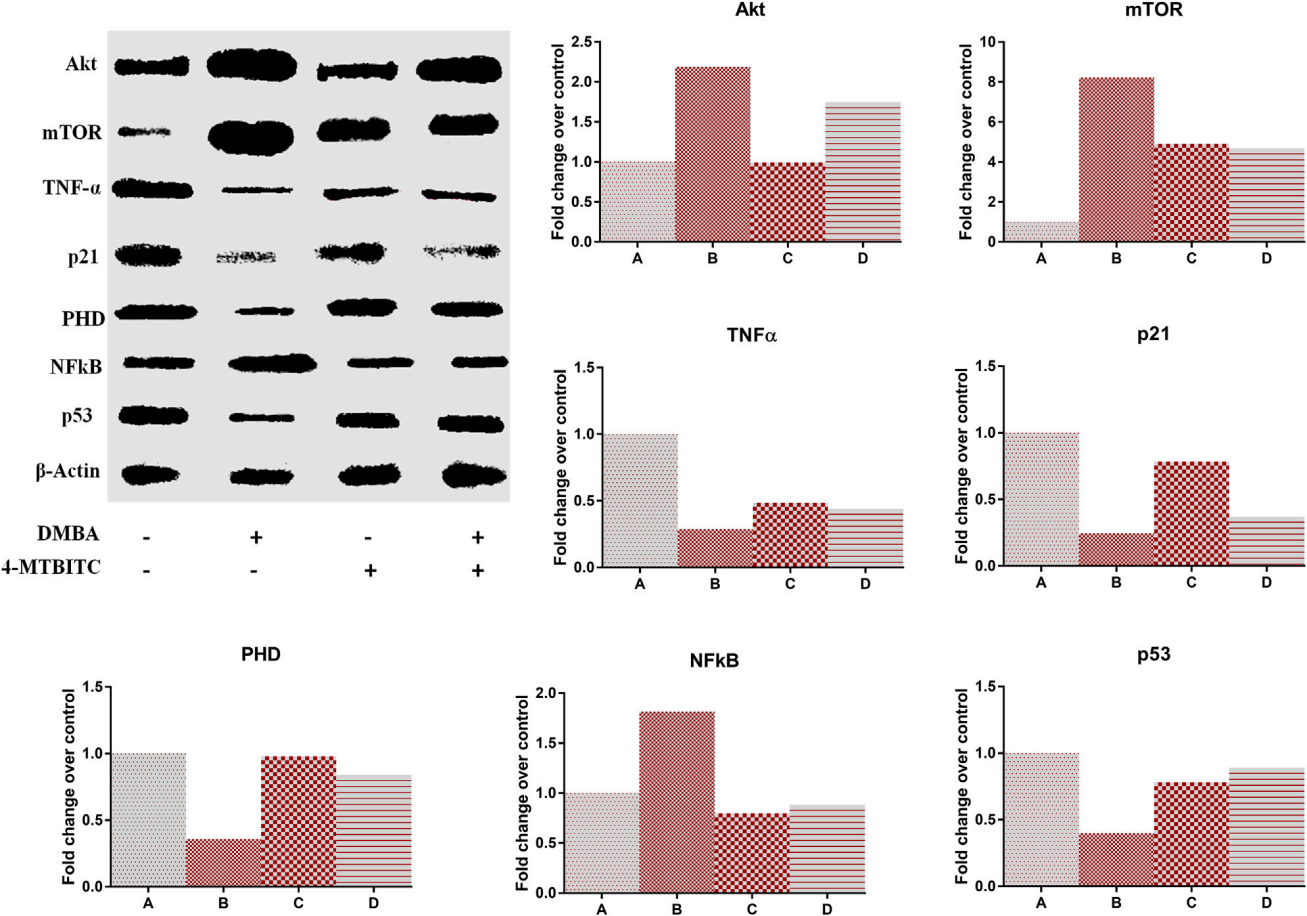
Western blot analysis of different protein markers in response to the treatment of 4-(methylthio)butyl isothiocyanate and DMBA. **(A)** Representative expression of protein markers, **(B)** Akt, **(C)** Mammalian target of rapamycin, (D) Tumor necrosis factor-α, **(E)** p21, F Prolyl hydroxylase, **(G)** Nuclear factor-kB, **(H)** p53.

The protein expression of Akt, mTOR, TNF-α, p21, PHD, p53, and NF-kB was evaluated using western blot analysis with normalization against Actin (Figure 6). DMBA induced the overexpression of cell proliferation markers Akt (119%), mTOR (722)%, and NF-kB (81%) in group B as compared to group A which is then lowered (20, 43 and 51% respectively) by the treatment of 4-MTBITC in group D as compared to group B (Figures 6B,C,G). Low expression of tumor suppressor proteins p21 (75%) and p53 (60%) was observed following the treatment of DMBA (Figures 6E,H). Counteraction of 4-MTBITC was seen to elevate the expression of p21 by 54% and p53 by 122%. TNF-α an inflammatory cytokine responsible for apoptosis/necrosis was seen decreased by 72% in group B compared to group A while increased by 57% following the treatment of 4-MTBITC in group D (Figure 6D). PHD, responsible for oxygen-dependent degradation of HIF-1α subunit by forming a complex with VHL was seen decreased by 65% following the treatment of carcinogen (group B) compared to group A (Figure 6F). Treatment of 4-MTBITC in group D inhibited the degradation of PHD and restored the protein expression by 133% as compared to group B.

### Histopathology

**FIGURE 7:**
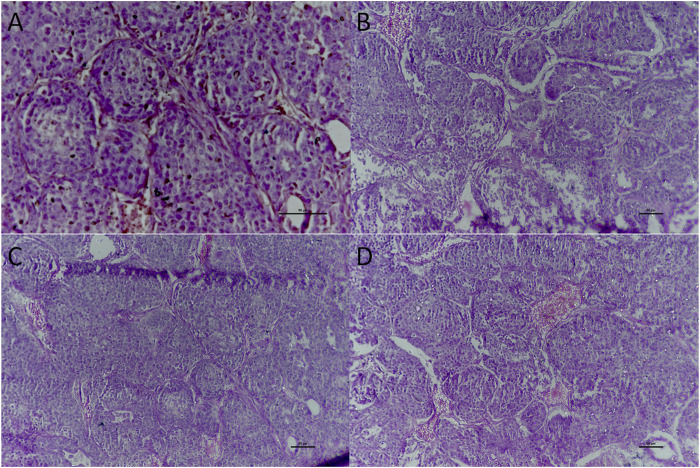
Histological examination of the mammary gland tissue of studied groups. **(A)** Group A x400 treated as control; **(B)** Group B x200 treated with DMBA; **(C)** Group C x200 treated with 4-(methylthio)butyl isothiocyanate; **(D)** Group D x200 treated with DMBA and then 4-(methylthio)butyl isothiocyanate.

Mammary tissue was sliced and washed with ice-cold PBS. Examination of group A showed dense collagenous tissue, blood vessels, and islands of glandular tissue surrounded by dense fibrous and adipose tissue (Figure 7). Administration of rats with DMBA (group B) revealed a neoplastic growth made up of trabeculae, ducts, and nests of malignant ductal cells. The stroma was desmoplastic and infiltrated by lymphocytic infiltrates mainly lymphocytes. In addition examination of mammary gland tissues of rats treated with 4-MTBITC after DMBA (group D) administration showed low infiltrating components.

## Discussion

Chemoprevention is the use of plant, synthetic, or biological chemicals to prevent, kill, or reverse the progression of premalignant cells from their initial stages to invasive disease. Mammary tumor induction by DMBA is one of the most popular methods for researching different aspects of breast cancer among various animal models (Barros et al., 2004). The present study was conducted to evaluate the modulation of hypoxia and glycolytic pathways as anti-breast cancer activity of 4-MTBITC in DMBA induced rats. The development of ROS and the peroxidation of membrane lipids in DMBA-induced mammary carcinoma-bearing animals resulted in a substantial rise in tumors (Davis and Kuttan, 2001). Increased antioxidant levels protect cells from oxidative damage while preserving regular structural and biochemical processes. The ubiquitous enzymes cytoplasmic SOD protect cells from free radicals generated during carcinogen metabolism. 4-MTBITC therapy resulted in a substantial rise in the rate of SOD enzyme, showing that 4-MTBITC has free radical scavenging activity by its stimulation. Breast cancer-bearing rats had higher levels of mammary lipid peroxidation products, which play a role in tumor development by combining with DNA to form MDA–DNA adducts, which cause genetic changes and contribute to cancer. Reduction in the levels of MDA content following 4-MTBITC treatment was very likely due to its anti-lipid peroxidative properties. GSH is an important antioxidant that breaks the cycle of a free radical movement to prevent the peroxidation of polyunsaturated fatty acids which can act as a carcinogenesis promoter (Adly, 2010). We observed that the level of GSH was low in DMBA treated rats which were significantly upregulated in 4-MTBITC treated cancer-bearing rats. Hepatic profile screening revealed the injury caused by an oral dose of DMBA which was further lowered with the treatment of 4-MTBITC. Before performing this anticancer study we performed a 14 days single dose acute toxicity study (OECD-423) from which the LD50 was came out to be 500 mg/kg. We then performed long-term toxicity studies using 4-MTBITC with different concentrations and it was found that the daily doses of 4-MTBITC for 28 and 90 days caused serious hepatic injury specifically to the female rats. We also performed pharmacokinetic studies which revealed its slow elimination (Ke = 0.0036 min^−1^) from blood plasma and hence in the current study, the frequency of dose was lowered to reduce such side effects (Unpublished data).

Tumor formation, development, angiogenesis, and metastasis are also thought to be influenced by inflammation. The main molecular players in the inflammation-to-cancer axis are proinflammatory mediators such as TNF-α and IL-6. TNF-α, an inflammatory cytokine that is strongly expressed in breast carcinomas, was reduced by 4-MTBITC. The inducible transcription factor Akt, mTOR, p21, and p53 regulates the expression of several genes involved in inflammation, proliferation, and apoptosis. 4-MTBITC inhibited the expression of genes NF-kB, and IL-6, which control the immune response to infection. It also inhibited the expression of cell proliferative markers such as Akt, mTOR, p21, and p53.

A high level of plasma leptin and a low level of adiponectin was observed following the DMBA administration. Adiponectin represses proliferation in breast cancer cells through inactivation of p44/42 MAPK protein 1 and 3 expressions, a stimulation of AMPK activity by phosphorylation at Thr172, and a decrease in Akt phosphorylation (Thr308) associated with an increased expression of LKB1 leading to a reduction of mTOR activity as evidenced by reduced phosphorylation of S6K (Taliaferro-Smith et al., 2009). Leptin induces the PI3K/Akt survival pathway by activating the phosphorylation of Akt Thr308 or Akt Ser473 and by stimulating the protein expression of PKC-alpha, or leptin activates the MAPK pathway by inducing ERK1 and ERK2 phosphorylation, or leptin rapidly and directly stimulates the STAT3 pathway and the up-regulate c-myc (one target gene of STAT3), at both mRNA and protein levels (Binai et al., 2010). As compared to DMBA treatment the levels were significantly reverted to normal with the treatment of 4-MTBITC.

There are multiple mechanisms by which HIF-1 mediates adaptive metabolic responses to hypoxia, such as increased glycolytic flux and decreased TCA flux, to minimize mitochondrial ROS production. HIF-1α a prime regulator of cell signaling in the absence of oxygen was upregulated in cancer-bearing rats. PHD an important gene that regulated the expression of genes involved in the hypoxia pathway was seen downregulated in cancer-bearing rats. In normal oxygen levels, glucose converts to pyruvate and enters the TCA cycle for the synthesis of ATP. As the oxygen level lowers the glucose converted to pyruvate will not enter the TCA cycle moreover lactate will be produced to lower the free radical formation from the electron transport chain. This lactate will serve as an instant energy source for neighboring cells. Lactic acid produced from lactate helps cancer cells to dissolve the extracellular matrix and finally metastasize to other organs. HIF-1α represses succinate dehydrogenase an important TCA enzyme that can limit the content of mitochondrial oxaloacetate required for aspartate biogenesis (Meléndez-Rodríguez et al., 2019).

Gluconeogenesis is the reverse of glycolysis through which glucose can be generated from a non-carbohydrate source. FBPase is a rate-limiting key enzyme that converts fructose-1,6-bisphosphate to fructose-6-phosphate. Loss of FBPase contributes to the development, promotion, and progression of multiple cancers, including basal-like breast cancer, whereas FBPase functions as a tumor suppressor by increasing oxidative phosphorylation and ROS levels, which are harmful to cancer cells (Dong et al., 2013). The activity of FBPase was seen enhanced with the treatment of 4-MTBITC as compared to cancer-bearing rats.

PPP pathway is critical for oxidative stress and the synthesis of nucleotides in the metabolism of breast cancer cells. 6PGD is a central enzyme in PPP’s oxidative branch that is active in nucleotide biosynthesis and redox status maintenance. DMBA induced the overactivation of this enzyme to withstand the increased requirement of nucleotides for proliferative cells which then reduced following the 4-MTBITC.

Amino acids are readily measured in blood, saliva, and urine, they may be useful biomarkers for diagnosis and screening during treatment. Serine is synthesized from glycolysis intermediate 3-phosphoglycerate through several enzymatic reactions. Serine is also involved in the production of antioxidants, which help cancer cells survive in low-oxygen conditions. The removal of serine causes breast cancer cells to proliferate less, an effect that can be entirely reversed by reintroducing it. Out of other amino acids, glutamate is the one that is consumed by cancer cells at the highest rate. Glutamic acid is the precursor of glutamine converted by glutaminase synthetase.

Blockage of arginine uptake results in apoptotic cell death in cancer cells (Abdelmagid et al., 2011). Nitric oxide is another metabolic consequence of arginine metabolism. Nitric oxide promotes angiogenesis and limits host immune response to promote tumor growth (Choudhari et al., 2013). Alanine is another important amino acid that is synthesized from pyruvate in the mitochondrial matrix (Groen et al., 1982). It is therefore expected that in hypoxic conditions more alanine is synthesized from pyruvate by competing with the pyruvate dehydrogenase. Recent studies suggest that the activity of aminotransferases is required for the formation of extracellular matrix in metastatic breast cancer cells (Elia et al., 2019). Asparagine is known as an amino acid exchange factor that regulates mTOR activation, nucleotide synthesis, proliferation, and metastasis (Knott et al., 2018). Asparagine is a metabolic dead end and may therefore be more available for exchange with extracellular amino acids (Krall et al., 2016). Along with other amino acids, asparagines preferably exchange with serine/threonine suggesting its indirect participation in energy production through the TCA cycle (Amelio et al., 2014; Krall et al., 2016). In the human body, glutamic acid is converted to glutamine by an energy-dependent reaction with ammonia using glutamine synthetase (Kulkarni et al., 2005). One of the most common amino acids in the human body is l-glutamine, a derivative of l-glutamic acid. Glutamine participates in the PPP pathway to withstand the high requirement of nucleotides by synthesizing purine and pyrimidines. It also transports to the mitochondrial matrix where glutaminase converts it into glutamate. This glutamate participates in the TCA cycle through conversion to α-ketoglutarate. Under hypoxic conditions, α-ketoglutarate can undergo reductive carboxylation to generate isocitrate which converts to citrate (Mullen et al., 2012). Citrate synthesis helps in lipogenesis. Amino acids are also helpful to boost the immune system. Tryptophan catabolism is known for its involvement in immune response modulation. The low plasma concentration of tryptophan in cancer indicates its rapid catabolism resulting in immunosuppression. This impairs the ability of dendritic cells and regulatory T cells to target and eliminate cancer cells (Greene et al., 2019). In the present study, amino acid profiling of serum-free plasma showed that the amino acids required to meet the high energy consumption for proliferative cells were affected by the treatment of 4-MTBITC. These alterations were confirmed by histopathological changes also.

Based on these, we speculated that the 4-MTBITC modulate DMBA induced glycolytic pathway and hypoxia pathway by downregulating the expression of HIF-1α and mTOR along with some amino acids that are needed for cancer growth.

## Conclusion

In conclusion, our study indicates for the first time that 4-MTBITC displays modulatory effect on DMBA induced key hypoxia-inducible pathway and metabolic pathway including glycolysis. It also modulates the expression of a central link of the proliferative pathway known as mTOR and Akt followed by modulation of p21, lactate production as well as amino acid metabolism involved in cancer cell proliferation. However, the direct interaction of 4-MTBITC with HIF-1α or glycolytic enzymes that may produce pharmacological response through *in-vitro* assay needs to be done. In-silico computational modeling may also help for further investigations.

## Data Availability

The original contributions presented in the study are included in the article/Supplementary Material, further inquiries can be directed to the corresponding author.

## Abbreviations

HIF-1α, hypoxia-inducible factor-1α; PPP, pentose phosphate pathway; DMBA, 7,12-dimethylbenz[a]anthracene; PAH, polyaromatic hydrocarbons; AhR, Aryl hydrocarbon receptor; ARNT, ahR nuclear translocator; ROS, reactive oxygen species; mTOR, mammalian receptor of rapamycin; ITCs, isothiocyanates; GSLs, glucosinolates; 4-MTBITC, 4-(methylthio)butyl isothiocyanate; SGOT, serum glutamic oxaloacetic transaminase; SGPT, serum glutamic pyruvic transaminase; SOD, superoxide dismutase; DTNB, 5,5′-dithiobis(2-nitrobenzoic acid); GAPDH, glyceraldehyde 3-phosphate dehydrogenase; PHD, prolyl hydroxylase; PTEN, phosphatase and tensin homolog; HSP-90α, heat shock protein-90α; FBPase, fructose-1,6-bisphosphatase; 6PGD, 6-phosphogluconate dehydrogenase; TNF-α, tumor necrosis factor-α; NF-kB, nuclear factor-kB; OPA, O-phthalaldehyde; MPA, mercapto propionic acid; FMOC, 9-fluorenylmethyl chloroformate; NBF, neutral buffered formalin; H&E, hematoxylin and Eosin; ANOVA, one-way analysis of variance; TCA, tricarboxylic acid cycle.
