# Supplementary material for: Modulatory Effect of 4-(methylthio)butyl Isothiocyanate Isolated From Eruca Sativa Thell. on DMBA Induced Overexpression of Hypoxia and Glycolytic Pathway in Sprague-Dawley Female Rats
Source: Front Pharmacol. 2021 Aug 10;12:728296. doi: 10.3389/fphar.2021.728296 (PMC8383164; doi:10.3389/fphar.2021.728296)
Supplement: Supplementary file 1 [file Table1.docx]

Supplementary Table S1: Primers used in the study were as follows:

| **Target gene** | **Accession No** | **Forward sequence** | **Reverse sequence** |
| --- | --- | --- | --- |
| HIF-1α | NM_024359.1 | GCAACTGCCACCACTGATGA | GCTGTCCGACTGTGAGTACC |
| Prolyl hydroxylase | XM_017590462 | CAGAATCACGAGACTGGAGCG | CAGAGACCTGCTAGGCTGAGG |
| Hexokinase | NM_022179 | TTGGTCACTCGTGTCGCTTG | CCGTGGGTTTCTTTGGGAGT |
| Pyruvate kinase | XM_006232593 | TTGCAGACACAGTTCCACGC | TCAGTTGAGCCACACTCGCA |
| Phosphoglucose isomerase | NM_207592 | ACCCTTCATTCTGGGAGCAC | GAGTCATGGGAGGTTACGGC |
| Aldolase | NM_012496 | GCACGTCACCCAGTAGTCTC | TCTCCATCCTTCCCTTCCGA |
| Triosphosphate isomerase | XM_003750654 | TCTGTGACTGGAGCGACTTG | GCAGGAAGGTAGGGGGATGA |
| GAPDH | NM_017008.4 | AGTGCCAGCCTCGTCTCATA | AACTTGCCGTGGGTAGAGTC |
| Phosphoglycerate kinase | NM_053291 | CACAGCATCTCAGCTCGTCT | ACCAGAGGCTACATACAGCG |
| Enolase | NM_012554.3 | TTGCACCTGCTCTGGTTAGC | AAGTCGGCAATGTGACGGTA |
| Phosphoglycerate mutase | NM_053290 | CGTGCGTAAAGCCATGGAAG | GGAGCCACAGGTCCCTATCT |
| mTOR | NM_019906 | TGCCAACTACCTTCGGAACC | CATGTCTCCGGCCCTCATTT |
| Succinate dehydrogenase | NM_130428 | AGCCTCAAGTTCGGGAAAGG | CACAGTGCAATGACACCACG |
| HIF-1β | NM_023090 | AGTTGGAAAGCCGGAAGACG | CTGATTGCCCACAGACCACT |
| PTEN | NM_031606.1 | ACCAATGGCTAAGTGAAGACGA | CAGGGCCTCTTGTGCCTTTA |
| HSP-90α | NM_001004082.3 | AGCTCAAGGAGTTCGATGGC | GGGGGAAGACACAAGCCTAT |
