# Supplementary figures and images for: Modulatory Effect of 4-(methylthio)butyl Isothiocyanate Isolated From Eruca Sativa Thell. on DMBA Induced Overexpression of Hypoxia and Glycolytic Pathway in Sprague-Dawley Female Rats
Source: Front Pharmacol. 2021 Aug 10;12:728296. doi: 10.3389/fphar.2021.728296 (PMC8383164; doi:10.3389/fphar.2021.728296)

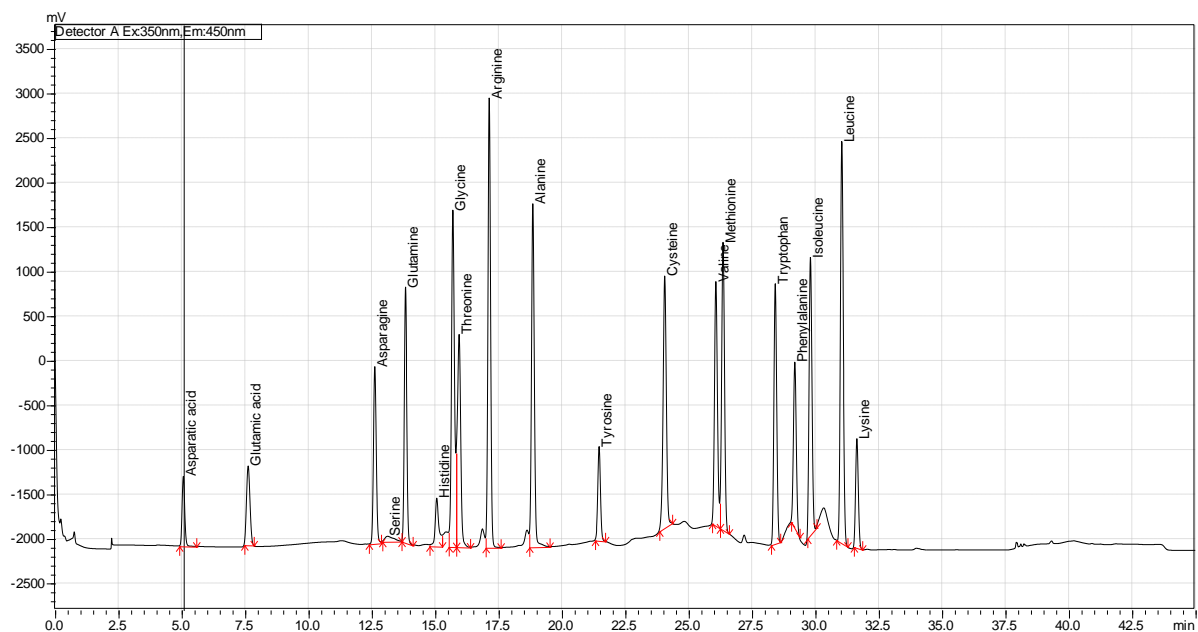

Supplement: Supplementary file 2 [file Image1.pdf]
